# The Protection of Civilians and ethics of humanitarian governance: beyond intervention and resilience

**DOI:** 10.1111/disa.12335

**Published:** 2019-03-01

**Authors:** Kristoffer Lidén

**Affiliations:** ^1^ Senior Researcher, Peace Research Institute Oslo, Norway

**Keywords:** ethics, global governance, humanitarianism, intervention, pluralism, protection, solidarism

## Abstract

The principle of the Protection of Civilians (PoC) in armed conflict has ethical repercussions in various actions undertaken by states and international organisations, from humanitarian relief, development aid, and peacekeeping, to warfare and military intervention. While the ethics of humanitarian intervention are instructive in this regard, most PoC practices should be conceived rather as modes of humanitarian governance across borders—from interventionist to resilience‐oriented kinds. The consequences of this for the ethics of PoC are explored in this paper, highlighting questions of power, culture, and complicity. By relating these questions to the ethical strands of solidarist and pluralist internationalism, it positions the ethics of PoC within the broader field of the ethics of world politics. Examples are drawn from recent scholarly debate on PoC efforts in war‐torn countries such as South Sudan. This analysis of the ethics of PoC reconfigures central positions in the debate on humanitarian intervention to an era of global humanitarian governance.

## Introduction

‘You are totally off the mark’, Hilde F. Johnson replied to Professor Mahmood Mamdani, when he blamed her in an interview for contributing to civil war and failing to protect civilians in South Sudan (Du verden!, 2016). Johnson was Special Representative of the Secretary‐General (SRSG) and head of the United Nations (UN) Mission in South Sudan (UNMISS) when war broke out in late 2013, and had been central to the preceding mediation and peacebuilding efforts (Johnson, 2016). Mamdani had been part of the African Union (AU) Commission of Inquiry on South Sudan, where he wrote his own ‘dissensus report’ (Mamdani, 2014). The gap between their views reflects a core problem of the UN agenda on the Protection of Civilians (PoC): extremely disparate views about its political potential and scope (Willmot et al., 2016). Mamdani takes as his point of departure the question of what would have been the correct political path to advance peace and protect civilians in South Sudan. Johnson, differing, focuses on what might be done in a setting where political solutions could not be imposed: ‘Mamdani is overestimating Western countries’ ability to trump and impose their solutions on African leaders’, she argues (Du verden!, 2016). Their disagreement is symptomatic of the political territory that international efforts of PoC operate in, stretched out between ideals and reality, ambitions and shortcomings, theory and practice.

While the doctrine of the Responsibility to Protect (RtoP) focuses on the responsibilities of states and international organisations to prevent and stop mass atrocities like genocide, war crimes, ethnic cleansing, and crimes against humanity, the closely related PoC doctrine focuses more broadly on the protection of individuals in situations of armed conflict, primarily civil war (on this connection between PoC and RtoP, see e.g., Breakey, 2012b; Welsh, 2016). In the ‘Aide Memoire’ of the UN Office for the Coordination of Humanitarian Affairs (OCHA), where UN policy on PoC is spelled out, PoC is defined by a number of ‘areas of concern’ reflected in UN Security Council Resolutions. These include displacement; humanitarian access, safety, and security of humanitarian workers; conduct of hostilities; small arms and light weapons; compliance, accountability, and the rule of law; media and information; and a series of specific protection concerns regarding children and women affected by armed conflict (OCHA, 2016, pp. 7‐29). The first, overarching concern of PoC is defined as ‘protection of, and assistance to, the conflict‐affected population’ (OCHA, 2016, p. 8). As such, it overlaps with the general objective of humanitarian protection in war‐torn countries (ICRC, 2008) and connects it with a broader range of actors and measures than those traditionally undertaken by humanitarian organisations.

As reflected in the debate between Mamdani and Johnson, international efforts to protect civilians are subject to extensive normative contestation in public debate and scholarly literature. Yet there is no systematic account of the ethics of PoC. When PoC is addressed in the literature on international ethics it is associated with the ethics of humanitarian intervention (i.e. military intervention for supposedly humanitarian purposes) (Hehir, 2013; Wheeler, 2000). However, this debate does not really cover the nature and justification of most practices of PoC. With rare exceptions, PoC is more about humanitarian assistance, international humanitarian law, peacekeeping, diplomacy, and the work of states and international agencies in related fields like development and security. Tying these activities together as aspects of a common humanitarian political agenda, PoC can be described as a mode of *humanitarian governance* (Barnett, 2010; Barnett, 2013; Fassin, 2011). Instead of outright foreign interference, humanitarian governance usually implies strategic partnerships between international and local actors in contexts of political struggle (Hameiri, Hughes, and Scarpello, 2017).

In this article, the ethics of PoC as humanitarian governance are investigated by analysing the theoretical underpinnings of contending normative positions on PoC. When people for instance disagree on how civilians in Syria should be protected (e.g., by multilateral intervention, a pragmatic peace deal, unilateral military support, or mere humanitarian assistance, etc.), it reflects different understandings not only of the Syria crisis but of the nature and morality of world politics. In the first section, the theoretical foundations of PoC in UN doctrine are introduced, and I show how UN policies are currently torn between the broad (interventionist) and narrow (resilience‐based) conceptions of PoC reflected in the debate between Mamdani and Johnson: ‘broad’ PoC as a comprehensive political agenda of global governance, addressing the root causes of conflict and building the institutional capacity needed for containing war; and ‘narrow’ PoC as a humanitarian agenda outside regular politics, concentrating on supporting local capacities for remedying the harmful effects of armed conflict. In the second section, this debate is situated within the ethics of world politics. First, it is shown to cover a more complex combination of ethical questions of power, culture, and complicity than indicated by existing debate on the scope of PoC. Next, these questions are related to positions of *political realism, internationalism*, and *cosmopolitanism*. Here, I suggest that while the ethics of humanitarian intervention often involve disagreement between realist and cosmopolitan positions, the debate on humanitarian governance primarily reflects different internationalist positions on a continuum between *solidarism* and *pluralism*. Against this backdrop, questions of power, culture, and complicity are explored in separate sections. The sources of political contestation about PoC are thereby clarified beyond simple dichotomies like humanitarian vs. political and sovereignty vs. intervention. The lack of such clarity currently mars scholarly and political debate on PoC, resulting in overly idealistic and critical positions. This analysis of the ethics of PoC therefore reconfigures central positions in the debate on humanitarian intervention to an era of global humanitarian governance.

## The Ethos of the Protection of Civilians

‘A United Nations that serves not only states but peoples—and becomes the forum where governments are held accountable for their behaviour toward their own citizens—will earn its place in the 21st century’ (Annan, 2012, p. 372). This final sentence in Kofi Annan's memoirs about his time as UN Secretary‐General reflects a view that has been manifested in a range of UN initiatives to promote development, human rights, and democracy since the 1990s. The concept of human security was symptomatic of this approach, challenging the idea of state security as an aim in itself and military power as the alpha and omega of security (UNDP, 1994; Owen, 2004; UN, 2004). Not only were states seen as secondary to individuals as security concerns; states were also complemented by civil‐society organisations and private corporations as actors of a global governance apparatus for the achievement of UN objectives (CGG, 1995; UN, 2014; Weiss, 2013).

Harmonising with this picture, the principle of PoC has been integrated into a broad array of international responses to armed conflict since its adoption by the UN Security Council in 1999: from peacekeeping missions, humanitarian relief efforts, and conflict mediation initiatives to continuous efforts at prosecuting the gravest violations of the principle in war‐crimes tribunals and the International Criminal Court (ICC). Reflecting this diversification of actors and agendas, Hugh Breakey distinguishes between four types of PoC: ‘combatant PoC’, regulating warfare; ‘humanitarian PoC’, responding to civilian suffering caused by armed conflict, including refugee management; ‘peacekeeping PoC’, introducing protection as a key aspect of peacekeeping mandates; and ‘Security Council PoC’, mandating responses to armed violence, including sanctions, and maintaining an overarching role in coordinating international PoC efforts (Breakey, 2012a, pp. 43‐57). To these four types, ‘state PoC’ should be added, as states have primary responsibility in UN PoC doctrine for taking necessary measures to ensure the protection of civilians within their territory. Furthermore, mediation involves a PoC dimension when seeking to halt violence against civilians. So does counter‐terrorism, given the prevalent targeting of civilians in terrorist attacks (Karlsrud, 2018).

The introduction by PoC of an explicitly humanitarian rationale to spheres not previously conceived as such is symptomatic of the dramatic expansion of humanitarianism since the 1990s (Barnett, 2011; Zesgin and Dijkzeul, 2015). This development has been defined as the emergence of ‘humanitarian governance’ as a significant political force in world politics, involving a ‘humanitarianisation’ of ‘global governance’ (Barnett, 2010, p. 3; Barnett, 2013). In *Humanitarian Reason*, Didier Fassin (2011, p. 6) describes how it is not just international organisations and the foreign policies of states that engage in modes of humanitarian governance, but that regular politics have been transformed through a humanitarian discourse—also in Western countries.

The scope for addressing the concerns of PoC can be interpreted broadly as part of a new *interventionist* agenda of humanitarian governance across borders, or more narrowly as a way of regulating existing practices of warfare and international engagement in accordance with the ideal of *resilience*—that is, of building local capacities for handling crises without foreign intervention (Williams, 2013; Chandler, 2012). The two approaches share a humanitarian objective, but differ about how to achieve it. The broad, interventionist concept seeks to use explicitly political means along the lines of the RtoP agenda, siding with those actors and institutions that harmonise with the objective at the international and domestic levels. The narrow, resilience‐oriented concept entails measures defined by the humanitarian principles of humanity, neutrality, impartiality, and independence, and seeks to carve out an extraordinary humanitarian space for protection in global politics on these grounds (on these humanitarian principles, see Slim, 2015). The narrow concept can still involve actors not generally considered ‘humanitarian’, like states and intergovernmental organisations, but limits the methods that these actors apply when acting in the name of PoC.

Relatedly, there is strong disagreement on whether humanitarian and development policies should be integrated, as in the broad concept of PoC—as proposed in the *Agenda for Humanity* adopted at the 2016 World Humanitarian Summit—or kept separate in order for humanitarianism to maintain independence from political agendas (for an example, see Lie, 2017). Underlying this is a tension that has characterised humanitarianism since its very beginning, between what Michael Barnett calls ‘emergency’ and ‘alchemical’ variants:

*Emergency humanitarianism concerns the provision of relief to those in immediate peril; cleaves to the principles of neutrality, impartiality, and independence; and has a hands‐off attitude toward politics. […] Alchemical humanitarianism involves saving lives at risk and addressing the root causes of suffering; operates with a less binding set of principles; and treats politics as a necessary and at times even welcome feature of humanitarian action* (Barnett, 2011, pp. 37, 39).

Variants of the two concepts of PoC are found in scholarly literature, organisational policies, and, importantly, in the foreign policies of states. Since its introduction to the UN Security Council, the veto‐wielding powers have been divided on the issue (SCR, 2015; Welsh, 2013). In resolutions and thematic debates in the Security Council, France and the UK have tended to take a broad approach, seeing PoC as key to the transformation of international law in a direction where state sovereignty is premised on human rights. Worried by how such ‘interventionism’ can privilege Western powers, Russia and China have defended a narrow approach where state sovereignty remains sacrosanct. The US has alternated pragmatically between the two, using protection language as part of the justification of international sanctions and interventions, while neither binding itself to the universal enforcement of the principle, nor to accepting humanitarian interventions that go against its interests (SCR, 2010, 2015, 2016; Lidén and Reid‐Henry, 2017; Bellamy, 2011; Adler‐Nissen and Pouliot, 2014). The extent to which strategic calculations influence the normative positions of the veto powers indicates how the ethics of PoC contain a deeply political dimension.

## PoC and the ethics of world politics

Judging by gruesome accounts of war in daily news and alarming reports from humanitarian organisations, the promise of PoC is in peril (e.g., Hartberg et al., 2015). The high toll on civilians in Syria, as well as in places like Iraq, Yemen, South Sudan, and Nigeria, have called the ability of the UN to uphold the principle into question. Yet, according to statistical evidence, the number of battle deaths, including civilian casualties, has steadily declined since its peak in 1950, although the war in Syria has caused a limited setback (Dupuy et al., 2017, Figure 3; HSRP, 2013). Although it is hard to prove the consequences of international PoC efforts on a counterfactual basis, studies of the impact of peace operations with a PoC mandate indicate a reduction in the number of civilian casualties (Hultman, 2013; HSRP, 2005; Hegre, Hultman, and Nygård, 2015).

While the objective of PoC is rarely questioned, the associated measures and their effects are subject to repeated criticism. Reflecting the domains where PoC policies are pursued, the PoC agenda is criticised for being severely overstretched, but also for not being sufficiently comprehensive (Ferris, 2011; Shesterinina and Job, 2016; Bradley, 2016; DuBois, 2010). For instance, efforts to enforce PoC for the regulation of warfare are characterised both as too limited and inconsistent and as too insistent. Military interventions in Afghanistan and Iraq have been criticised for appropriating and manipulating humanitarian relief, but also for not prioritising it enough. Humanitarian relief to refugees and victims of war is criticised both for favouring governments (for instance, when governments channel relief to their supporters and to areas they control), and for feeding rebels and granting them safe havens in refugee camps at the expense of sovereign authorities. Peacekeeping operations with a PoC mandate are criticised for being too aggressive and interventionist, but also for being too limited and weak. The military intervention in Libya was criticised for abusing the principle of PoC for the purpose of regime change, while a lack of intervention for regime change in Syria has been criticised by reference to the same principle.

These criticisms bring up a series of ethical questions that can be summed up as: (i) *Power:* is it justifiable to advance the power of certain actors for the sake of PoC?; (ii) *Culture:* is it justifiable to promote certain norms and ideas through international engagement for the sake of PoC?; (iii) *Complicity:* is it justifiable to be complicit to harm for the sake of PoC? (this typology is inspired by the introduction to humanitarian ethics in Slim, 2015; see also Löfquist, 2017; Givoni, 2011a, 2011b). As demonstrated below, answers to these questions typically fall between the broad and narrow concepts of PoC.

Questions of power, culture, and complicity are central not only to the ethics of PoC but to the ethics of world politics in general. At this level, they might be reformulated as: (i) *Power:* is it right for powerful states to organise world politics in accordance with their own interests and morality, or should politics be based on an equal distribution of power among the world's population? (ii) *Culture:* should the world's cultures be regulated by universal moral standards like human rights—involving a minimum cultural consensus—or ought cultures to be sheltered from such a civilising mission, making the independence of cultures an ultimate moral objective in itself? (iii) *Complicity:* assuming the current world order is not in harmony with the answers to the above questions—should the problems be addressed through nonideal but realistic revisions to the order (thereby accepting a degree of complicity by reinforcing it), or should the order be resisted in pursuit of more revolutionary change (accepting that it is less efficient and realistic, at least in the foreseeable future)?

The strands of *political realism, internationalism*, and *cosmopolitanism* present us with different answers to these questions (for an overview of these positions, see e.g., Brown, 2002; Caney, 2005; Dower, 2009). Crudely speaking, realists see international politics as an anarchic sphere of conflict, and reject the idea of shared morality across nations. They therefore (i) think it necessary for the strongest states to resolve the world's problems according to their own interests, and (ii) that it is up to states to protect and promote their own cultures and moralities. They therefore (iii) see the world as being in harmony with their attitudes to questions of power and culture, and therefore see no problem of complicity. Internationalists disagree, and (i) see international collaboration between equally sovereign states as a precondition for resolving shared problems. While involving a thin moral and cultural consensus, this (ii) bodes for a high degree of cultural diversity within and across states. Concerning (iii) complicity, internationalists see no realistic alternative to the current world order and ascribe the responsibility for solving the world's problems to the state. Cosmopolitans, on the contrary, understand world politics to be constituted not by states but by individuals. (i) Power should be distributed equally among all ‘citizens of the world’, and (ii) cultural diversity should be rooted in moral universalism; (iii) shared problems should be resolved by the best global set of political institutions possible, and they see the state system as falling short in this regard. To cosmopolitans, both internationalist and realist solutions are therefore complicit in harm when reinforcing the current world order.

The internationalist position leaves room for extensive variation, from a *pluralist* rejection of any international interference in the internal affairs of states (e.g., Jackson, 2000) to a *solidarist* qualification of state sovereignty as relying on compliance with certain fundamental norms like basic human rights, implying extensive international collaboration for attaining these standards (Vincent, 1984; Wheeler, 2000; Rawls, 1999). As prescriptive doctrines, the narrow and broad concepts of PoC find support in these pluralist and solidarist variations of internationalism respectively (on the debate between these positions, see e.g., Wheeler, 2000, pp. 21‐33; Bull, 1984; Dower, 2009, pp. 52‐60; Buzan, 2004; Clark, 2007).

**Figure 1:**
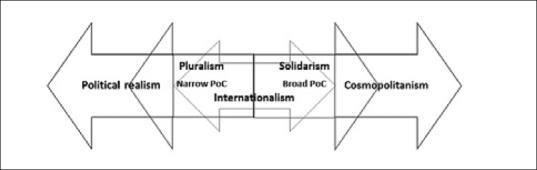
Situating PoC in the ethics of world politics **Source**: author.

As recently argued by Simon Caney, Barry Buzan, and Michael Walzer, among others, we should think of the relation between pluralism and solidarism as a continuum, or spectrum, with pluralism stretching towards realism and solidarism towards cosmopolitanism within internationalist confines (Caney, 2005, pp. 12‐13; Buzan, 2004, pp. 48‐50; Walzer, 2004; Lidén, 2014, pp. 31‐35). On this continuum, we may imagine a parallel continuum between the narrow and broad concepts of PoC (see Figure 1). Although involving international engagement in the internal affairs of states, the narrow concept of PoC is compatible with moderate forms of pluralism because it does not challenge the sovereignty of states. The broad concept of PoC has a cosmopolitan dimension, aspiring to revise the international order for the universal protection of individuals. However, like RtoP, it falls within the solidarist camp because it does not challenge the fundamental role of states in world politics, and instead entails a programme for adjusting a state‐based world order to the principle of PoC.

The debate between solidarist and pluralist positions on international intervention which developed in the 1990s centred on the legitimacy of military humanitarian intervention (Hehir, 2013; Wheeler, 2000). Although relevant to the debate on PoC, the ethics of PoC cannot be reduced to this question. Apart from rare but significant cases of military intervention, as in Libya and Kosovo, the contemporary politics of PoC unfold within the limits of the principle of state sovereignty in international law (see e.g., Curran, 2017). With international support given to states for building the capacity to protect the population, state sovereignty is also conceived as a central *source* of protection (Eberwein and Badie, 2006). Furthermore, international PoC efforts are never sheer manifestations of foreign decrees in practice, as argued in the next section. Even when motivated by a broad interventionist agenda, they manoeuvre within the social and political landscapes of target countries, exercising ‘the art of the possible’ within the confines of customary international law. Yet they have an impact—politically, economically, socially, and culturally, as reflected in the notion of humanitarian *governance*. While relating to questions of non‐intervention and self‐determination, we therefore need to avoid reducing the ethics of PoC to a derivation of the question of humanitarian intervention.

This criticism of the ‘intervention’ trope harmonises with the policy doctrine of ‘resilience’, which has been extremely popular in international organisations over the past decade (Chandler, 2014). Policies of resilience focus on building local capacities for handling crises instead of upholding dependency on international support (for the link to PoC, see e.g., Williams, 2013). This approach was strengthened by the shortcomings of international interventions and peace operations in the 1990s and 2000s, with a recognition of their reliance on local ownership and participation (Ginty and Richmond, 2013). However, if conceived as a locally driven (pluralist) alternative to (solidarist) international intervention, the degree of interference involved in internationally induced resilience is underestimated (Chandler, 2012). When conceptualising international PoC efforts as a mode of humanitarian governance, we therefore need to focus on both their international and domestic political dimensions, independent of whether they are conceptualised as interventionist or resilience‐based.

## The politics of humanitarian governance

The concept of global governance relates to the combination of international and domestic politics involved in humanitarian governance (Weiss, 2013). However, it is often used in ways that give a rather concerted and one‐directional impression of global political processes shaping the domestic affairs of states. Before categorising humanitarian governance as a mode of global governance, the role of political struggle and of local political actors in determining its conditions and outcome must therefore be acknowledged.

In *Regulating Statehood* (2010), Shahar Hameiri provides just such an alternative conception of global governance in the context of international peace operations (Hameiri, 2010). Using case studies from Cambodia, Solomon Islands, and East Timor, he demonstrates how responses to war and deprivation by international organisations are characterised by a logic of risk‐management on the part of their donors: the organisations manage threats to the international economic and military orders that regional and global powers rely on (Hameiri, 2010, pp. 40‐42; Clapton, 2014). It is not primarily norms like human rights that determine the character of their work, he argues, but their political interests and the practical knowledge among international experts and bureaucrats of how political problems like civil strife are to be handled within a state framework (see also Sending, 2015; Barnett and Finnemore, 2004). Although such interference can represent a challenge to the state system, Hameiri describes how the expertise presupposes the state to be a regulative ideal, somewhat filling the gaps of an imagined state apparatus. In effect, the operations do not formally replace the state apparatus or challenge the legal sovereignty of the intervened states, but transform, or ‘transnationalise’, parts of their governing institutions (Hameiri, 2010, p. 38). Instead of regarding these activities as extraordinary and transitional, he argues that they actually form a permanent and globalised form of transnational governance. This is in line with what Neumann and Sending argue in *Governing the Global Polity* (2014), where they see global governance as a continuation of liberal state governance rather than a replacement for it, relating to a broader trend of neoliberal globalisation (Neumann and Sending, 2014, p. 5).

In Hameiri's 2010 account, the domestic counterparts of international organisations—local politicians, bureaucrats, non‐governmental organisations (NGOs), village councils, corporations, etc.—nonetheless play an active role, both in forming the premises of the assistance and in using it in support of their own political and economic agendas. Their agendas tend not to be the same as those behind the assistance, resulting in compromises and new transnational political constellations (see also Richmond and Mitchell, 2011; Barnett and Zürcher, 2009). In a 2017 study, Hameiri, Hughes, and Scarpello analyse this negotiation of foreign assistance as a ‘politics of scale’ concerning the level at which political problems are to be managed—from the international to the very local—with significant consequences in terms of which actors and institutions are included and excluded, empowered and marginalised (Hameiri and Jones, 2017; Hameiri et al., 2017). The ability to take advantage of international intervention has become an important source of power and privilege in conflict‐ridden developing countries, replacing the co‐opting of strategic support from the contending superpowers during the Cold War.

Hameiri's perspective coincides with David Kennedy's position in *A World of Struggle* (2016). Rejecting instrumental notions of global governance as neutral responses to objective problems, he highlights how conceptions of the problems and their solutions result from competing claims to expertise. Experts represent different organisations, corporations, state agencies, disciplines, doctrines, and personal ambitions, and instead of merely translating authoritative knowledge or professional experience, they strategically position their claims (Kennedy, 2016, pp. 3‐4, 31). Perhaps this is also part of the reason why experts often fail to give advice in line with the official justification of humanitarian operations, as documented by Kennedy in a previous study (Kennedy, 2007). The lack of transparency and accountability in such expert advice reproduces hierarchies and advances vested interests in the name of humanity (Kennedy, 2016, Part 1). Sending (2015, p. 129) takes this perspective a step further by analysing the power dynamics which constitute expertise in the first place, and shares Kennedy's concern for a shortage of ‘publicness and legitimacy’ of expertise in global governance.

Hameiri's account of global/transnational governance also finds support in the definition by Dorothea Hilhorst and Bram J. Jansen of humanitarian action as constituted in a social arena where principles, vulnerability, rights, and services are appropriated by agencies and their beneficiaries to advance their own agendas in settings of severe competition (Hilhorst and Jansen, 2010). On the one hand, humanitarian agencies compete for funding and influence, and for this purpose they strategically position themselves as the most humanitarian, effective, informed, and well‐connected. On the other hand, aid recipients and other actors have far more influence on the allocation of services than is commonly acknowledged. Host governments, political parties, organisations, and local strongmen interfere with the allocations and programmes, and beneficiaries learn how to play the organisations. As Hilhorst and Jansen conclude:

*Processes by which actors define each other do not follow definitions of principles as such; they constitute political struggles in which discourses of humanitarianism and human rights act as major devices. […] By focusing on the everyday practices of aid it becomes clear how humanitarian headquarter claims to political neutrality and the application of universal normative values are negotiated through the micro‐physics of power in humanitarian arenas* (Hilhorst and Jansen, 2010, pp. 1136‐1137).

When evaluating the politics of PoC, it is therefore misleading to take the official objective of PoC as a given. The same goes for any alternative account of the motives of humanitarian governance, like risk governance, human‐rights promotion, economic self‐interest, etc. These are all relevant to explaining the practices, but the ethical significance of PoC efforts are not reducible to any one of them. Instead, we need to take the actual practices of PoC as the basis for ethical evaluation, considering their political nature and effects. Concentrating on the difference between solidarist and pluralist positions, the applied ethics of PoC conceived as practices of humanitarian governance will now be explored through the questions of power, culture, and complicity.

## Power

A recurrent theme in criticism of PoC‐related practices is that they are subject to political pressure, reinforcing global, regional, national, and sub‐national hierarchies (Slim, 2015, pp. 14‐15; Duffield, 2007; Kennedy, 2007). When seen from the perspective of PoC as transnational humanitarian governance, this observation comes as no surprise. The idea that efforts of PoC can be independent from political influence is unrealistic; this is equally true when it comes to humanitarian actors. The question, rather, is under what conditions and to what extent such ‘compromised’ governance is justified.

In an in‐depth study of international humanitarian organisations during armed conflict in Sri Lanka and the Philippines, Sreeram Chaulia argues that they form part of a military–humanitarian complex where civilian protection becomes a continuation of the warfare (Chaulia, 2011). The organisations studied did not primarily protect the lives of civilians, but provided shelter, food, and development in the name of protection, coinciding with the interests of one of the parties involved in the warfare. In Sri Lanka, for instance, the organisations ran government‐controlled detention camps for refugees from areas held by the Tamil Tigers (LTTE), and in the Philippines they collaborated closely with US forces directly involved in the Moro war. Chaulia concludes that the relief efforts followed a pattern where international humanitarian organisations ‘have not only been harnessed for partisan ends by donors, host states and violent non‐state actors, but also engendered through their own reserves of grants and projects a wholesale “NGOisation” of the Global South’ (Chaulia, 2011, p. 224; for a theoretical analysis of this form of governance, see Lidén et al., 2016).

Chaulia presents this as a devastating critique. Yet, from a solidarist perspective, it would be justified if harmonising with the right universal norms. When limited to the norm of protection from mass atrocities, the fact that PoC serves the interests of certain international or local groups is a necessary and justifiable side‐effect. To a ‘thicker’ *liberal* form of solidarism, the political side‐effects of protection ought to be in accordance with the advancement of human rights and democracy (Lidén, 2014, p. 27). From a pluralist perspective, however, bolstering the power of international actors and their local collaborators is ethically problematic because it undermines locally rooted political dynamics. A ‘thin’ pluralism—overlapping with ‘thin’ solidarism—may still allow for such negative side‐effects for the protection of civilians in the gravest cases of mass atrocities. A ‘thick’ pluralism, however, rejects such interference because it is expected to destabilise the international system and undermine more representative and durable locally driven reactions to the problems at hand (e.g., Jackson, 2000).

Because Chaulia avoids normative questions in his theoretical framework, it is not entirely clear where the negative normative evaluations of his empirical findings come from. However, in the conclusion and epilogue, he declares a normative commitment to ‘validating the dignity of civilians in war’, and refers to the fundamental aspirations of the UN Charter: life, liberty, and the dignity of the human person (Chaulia, 2011, pp. 220, 223). It is not the solidarist advancement of international standards that he laments, therefore. Instead, it is what he describes as the manipulation of this agenda by the great powers, reinforcing a ‘capitalist world‐system’ (Chaulia, 2011, p. 223). In opposition to a liberal version of solidarism, he pronounces a Marxist‐inspired socialist variation of solidarism, but observes that it is unrealistic to expect to achieve his vision within the current international humanitarian system (Chaulia, 2011, pp. 202, 221, 225).

Chaulia's analysis is an example of how criticisms of the power politics of PoC rely on normative theoretical presuppositions that often remain implicit. Similar examples of prominent pluralist and solidarist analyses of the question of power in humanitarian protection efforts could be investigated (for a magnificent source of examples, see for instance Magone et al., 2011). However, I will now turn to the related problem of culture in the ethics of PoC.

## Culture

The problem of culture is almost as prevalent in critical analyses of practices of PoC as the problem of power. A recurrent theme is that international actors cannot adjust to local norms, ideas, and institutions relevant to the aim of protection. Instead, it is contended, foreign moral, legal, political, religious, social, and economic models are transplanted into the host countries, with ‘neo‐colonial’ effects (Slim, 2015, pp. 10‐11). Again, this ethical problem is in line with the analysis of PoC as transnational humanitarian governance. According to the accounts by Hameiri, Kennedy, and Sending, however, the problem is not so much a normative commitment to foreign ‘culture’ or unwillingness to recognise the ‘local culture’, as one of biases built into practical knowledge of what the political problems of PoC are and how they ought to be addressed. There is no lack of emphasis on taking local culture into account at the level of policies, strategies, and guidelines. The problem is rather how these conditions are interpreted and transformed into practical solutions. This is also how the cultural dimension is wedded to power: as argued above, the norms, ideas, and identities of the ‘protectors’ emerge from, and reproduce, prevalent social systems like the modern (neo)liberal state. Not as a principled imposition of foreign culture, but as constitutive of who the actors that ‘adapt to local culture’ are, and how they think about such adaptation. Michal Givoni offers a telling example when documenting how the agenda of Médecins Sans Frontières (MSF) emerged from the professional identity of the doctors involved, positioning themselves in a French political context (Givoni, 2011b). By contrast, the story of MSF is usually told as a reaction to shortcomings in the humanitarian system through a more effective and uncompromising approach.

In a comparison of several case studies of international PoC efforts, Niels N. Schia, Benjamin de Carvalho, and Paul D. Beaumont (2016) highlight five problems all related to the problem of culture: (i) taking civilian identity for granted; (ii) the myth of the passive civilian; (iii) the sedentary bias of refugee management (the assumption that refugees naturally belong where they fled from); (iv) gender essentialism and protecting ambiguous groups; (v) the fallacy of *terra nullius*—ignoring existing capacities, traditions, and institutions. If we started from the policy rhetoric of PoC this would be a problem, because it hinders the effective protection of civilians and causes unforeseen side‐effects. In the context of PoC as humanitarian governance, however, the five flawed assumptions are not really assumptions but reflections of the conditions under which PoC efforts operate and the way the policy language of PoC is strategically employed. As with the problem of power, the question for ethics is not whether cultural biases are justifiable, but how to assess practices where cultural bias is necessarily involved.

To solidarists, the question of culture is how international norms can be translated most accurately to the local context, thereby anchoring international engagement in local culture. This is not a matter of moral relativism, but of maintaining the meaning of the norms in a different cultural setting. To pluralists, in contrast, the question is how local culture can be maintained in the face of foreign assistance, avoiding rationales like the rooting out of ‘backward’ or ‘brutal’ traditions and institutions in the name of humanitarian protection and human progress. Complicating this matter further is the futility of a homogenous notion of ‘local culture’ that international actors can promote or adjust to (Lidén and Jacobsen, 2016). In addition to a multiplicity of interests and political agendas, each host country of humanitarian governance involves a range of ‘cultures’—urban and rural, modern and traditional, etc.—involving different understandings of the problems at stake and of how they ought to be resolved (see e.g., Mamdani, 1996).

For instance, an analysis of the sedentary bias in Kenyan refugee policies reflects a collusion between, on the one hand, the various outlooks of international agencies, government officials, foreign countries, and Kenyan citizens, and, on the other, the multiple identities of the refugees (Horst and Nur, 2016). Slogans like ‘resilience’ and ‘local ownership’, therefore, do not themselves resolve the ethical problem of culture. International actors will still evaluate exactly what resilience and local ownership implies in a setting of struggle and competition, and their evaluations will be ‘compromised’ by their beneficiaries and local powerholders. Humanitarian governance, in effect, involves rather a meeting, or negotiation, between international and local cultures—in the plural. The ethical problem of culture for PoC is, therefore, not whether to privilege international or local culture, but how to open these categories for more nuanced understandings of the norms and ideas at stake, how they relate to the distribution of power among social groups, and how they ought to be dealt with in the context of international protection efforts. The traditions of pluralism and solidarism offer a host of insights into the principles involved in these questions, as does literature on concrete measures of humanitarian relief, IHL, peacekeeping, state‐building, sanctions, and mediation on their practical side. The challenge is how to combine these insights in a nuanced ethical debate on the cultural dimension of PoC.

## Complicity

The strongest criticism of PoC practices emerges when they are perceived as being complicit in wrongdoing (Lepora and Goodin, 2013; Anderson, 1999; Slim, 2015, pp. 18‐19). The bolstering of unjust hierarchies of power or hegemonic cultures may be seen as harmful—making questions of power and culture overlap with the question of complicity. However, debates on complicity typically centre on events that solidarists and pluralists alike would see as harmful, such as war or famine. The moral verdicts on such cases, nonetheless, tend to be contentious, where some see pragmatic engagement for minimising harm as complicity, while others see the same efforts as necessary compromises reflecting a heroic willingness to ‘get one's hands dirty’ in a good cause. Conflicting understandings of what the options are and what the consequences of non‐action would be often lurk in the background of such disagreements. In that respect, the perspective of transnational governance offers a basic corrective to debates where critics presuppose that PoC can be conducted outside the realm of politics, and where such criticism is rejected by reference to the fact that such humanitarian ‘purity’ is impossible—instead of having a nuanced debate on possible alternatives (Lepora and Goodin, 2013, p. 11).

In Mamdani's account of the war that broke out in South Sudan in December 2013, the preceding international mediation, state‐building, and peacekeeping practices are responsible for the crisis, together with the government (Mamdani, 2014, §175). He argues that by generating a political solution that relied on the power of the rebel group Sudan People's Liberation Movement (SPLM) and its army (SPLA), the eventual building of a new South Sudan rested on grounds that were too fragmented, military and undemocratic. The many factions of the SPLA had previously been fighting each other, and were divided along various lines, including ethnic affinities. To Mamdani, the eventual conflict within the SPLA should have been foreseen by the Troika (US, United Kingdom and Norway) that pushed for the Comprehensive Peace Agreement, and effective preventative measures to protect civilians should have been taken in response—including an insistence on a less militarised and more inclusive political transition and the deployment of a peacekeeping force capable of meeting its mandate of PoC. In similar fashion to failures in Rwanda and Srebrenica, the UNMISS peacekeepers did not intervene when violence erupted. Instead, they remained in their compounds and eventually succumbed to pressure from people fleeing to their gates in need of protection, resulting in ad hoc ‘protection of civilians’ camps. These camps were eventually drawn into the political dynamics of the conflict and created tensions between the people in the camps and residents in neighbouring areas (Mamdani, 2014, §105‐110).

Do these shortcomings make the international efforts ‘complicit’? Was there a better alternative to relying on the power of the SPLM—the uncontested powerhouse of South Sudan—and could the peacekeepers have averted warfare by taking up arms against the militias and government forces? In theory, the AU and UN Security Council could have exerted sufficient pressure on the SPLM to allow for a more democratic and representative political transition, and they could have devised much larger and better equipped peacekeeping operations with an even sharper mandate (which would again require strong pressure on the government). However, in practice there was insufficient support from both international and local political actors for the UN mission to effectively exert such pressure and commit sufficient resources, as documented by Johnson in her ‘untold story’ of the process (Johnson, 2016; see e.g., Chapter 4). The problem was not a lack of commitment to PoC as an ideal, but political limits to the engagement. Furthermore, even if the SPLM accepted a more inclusive and democratic transition, with early elections after independence, it would be virtually impossible to avoid conflict between powerful generals and politicians in the absence of effective enforcement mechanisms and judicial bodies. The UN operation relied on the support of a government enmeshed in political struggle, and breaking with the government would have entailed either withdrawal or coercive intervention—which would probably be even less conducive to the protection of civilians. Hence the conditions for international mediation and peacekeeping efforts, including their PoC dimension, resonate with Hameiri's account of the limits to peace operations.

In fact, Mamdani's account of the history of peace efforts carries all the hallmarks of Hameiri's conception of state regulation. He describes how international engagement since 2005 has been regulated by unrealistic objectives of state‐building ‘from scratch’ based on ‘best practice’ from different contexts. With the limited capacities of local actors to manage this process, international organisations were contracted as builders of what became a new country in 2011, and humanitarian and development agencies functioned as surrogate service‐providers (Mamdani, 2014, §92; Johnson, 2011, pp. 180, 184).

Pointing to the responsibility of the government and army, Johnson denies that the failure to prevent the gruesome violence that unfolded after 2013 when the political and military pillars of the new state structure collapsed makes her complicit, as the representative of international engagement. In her view, there was no realistic alternative to the peace agreement but continued warfare. The eventual peace‐implementation efforts balanced serving the local authorities and meeting international standards of ‘good governance’. In criticising these efforts, Mamdani's argument could be expected to reflect a pluralist rejection of international interference. However, his analysis instead manifests a call for a more coherent form of solidarism where international objectives are better adjusted to the local context. As he writes, such contextualism does not exclude the promotion of universal values: ‘Context is not the opposite of a universal value or standard. Context is an understanding that any concrete situation is an outcome of multiple causes: historical, political, moral, and economic’ (Mamdani, 2014, §173). It is the limited ability to recognise local conditions for such solidarist aspirations that he laments: ‘The call for a contextual understanding is an argument that we need to move away from a single‐formula prescription to understand the precise interaction of multiple processes in the creation of a single event or outcome’ (Mamdani, 2014, §173).

Johnson would probably agree with this statement in principle but would see alternative solidarist modes of international engagement as unrealistic. Having experienced the shortcomings of international support and the reliance on local elites for operating in the country, she has a different view of what a solidarist PoC agenda entails. In other words, a broad conception of PoC can imply rather limited measures if the room for PoC is thought too constrained for a broader solidarist agenda to succeed. Mamdani sees the situation differently, prescribing changes to the very political landscape wherein Johnson's state was built. We may therefore read the debate between Johnson and Mamdani as a disagreement not between solidarism and pluralism but between solidarist accounts of how broad PoC can realistically get. Again, the distinction between broad and narrow PoC turns out merely to be the first step towards a more nuanced understanding of the ethics of PoC.

## Conclusion

Too often, normative debate on PoC is caught in the rhetorical web of UN policies, leaving an impression of PoC as a concerted humanitarian response to problems defined by global political consensus. When they encounter undeniable deviations from this image, the policies are rejected as a scam or ridiculed for their deficiencies. Instead, the ethics of PoC should be rooted in a more adequate conception of humanitarian governance involving political struggle at all levels.

In effect, the applied ethics of PoC are not a matter of cosmopolitan vs. realist positions on humanitarian intervention, with cosmopolitans shelving state sovereignty for the sake of human rights and realists rejecting such idealism as naïve and counterproductive. Both of these positions are at play in the choices and dilemmas of contemporary practices of PoC, but only through their solidarist and pluralist interlocutors in internationalist ethics. Hence the applied ethics of PoC should concentrate on clarifying principled positions on concrete problems along a spectrum between pluralist and solidarist extremes.

The questions of power, culture, and complicity that have been raised in this paper provide a framework for such analysis. Through an exploration of these questions we have seen how the politically contentious question of the scope of PoC—of where PoC starts and ends—covers a range of nuances, making it unconvincing to derive a general (broad) interventionist or (narrow) resilience‐based recipe of PoC from a solidarist commitment to human rights or a pluralist rejection of international interference. Yet from our analysis of contemporary practices we may conclude that the broadest conception of PoC as a comprehensive doctrine of global governance is entirely unrealistic and therefore incompatible with a coherent solidarist position. Meanwhile, the narrowest conception of PoC as purely apolitical and consensual does not itself guarantee a pluralist outcome, as its realisation still relies on political interpretation in contexts of struggle. To facilitate a productive debate between solidarist and pluralist perspectives, policies of PoC should therefore be formulated both in narrower and broader terms than the current ethos of PoC in the UN: narrower in terms of its ends, focusing on the core humanitarian objective of protecting civilians (rather than a comprehensive political doctrine), and broader in terms of its means (rather than conceiving these as a neutral translation of its ends). In this manner, policies of PoC would invite ethical debate on their political implementation instead of leaving their operationalisation to experts, bureaucrats, and practitioners alone.

## Acknowledgements

The research for carried out within the project ‘Protection of Civilians: from principles to practice’, funded by the HUMPOL program of the Research Council of Norway. The author would like to thank colleagues in the Protection of Civilians project for input and collaboration, the editors of the special issue for excellent follow‐up, the anonymous reviewers for valuable comments, and the copy‐editor for a solid job.
